# Development and External Validation of a Nomogram Predicting the Probability of Significant Gleason Sum Upgrading among Japanese Patients with Localized Prostate Cancer

**DOI:** 10.1155/2011/754382

**Published:** 2010-11-07

**Authors:** Takashi Imamoto, Takanobu Utsumi, Makoto Takano, Atsushi Komaru, Satoshi Fukasawa, Takahito Suyama, Koji Kawamura, Naoto Kamiya, Junichiro Miura, Hiroyoshi Suzuki, Takeshi Ueda, Tomohiko Ichikawa

**Affiliations:** ^1^Department of Urology, Graduate School of Medicine, Chiba University, 1-8-1 Inohana, Chuo-ku, Chiba 260-8670, Japan; ^2^Division of Urology, Chiba Cancer Center, Chiba 260-8717, Japan; ^3^Department of Urology, Sakura Medical Center, Toho University, Sakura 285-8741, Japan

## Abstract

*Objective*. The aim of this study is to develop a prognostic model capable of predicting the probability of significant upgrading among Japanese patients. *Methods*. The study cohort comprised 508 men treated with RP, with available prostate-specific antigen levels, biopsy, and RP Gleason sum values. Clinical and pathological data from 258 patients were obtained from another Japanese institution for validation. *Results*. Significant Gleason sum upgrading was recorded in 92 patients (18.1%) at RP. The accuracy of the nomogram predicting the probability of significant Gleason sum upgrading between biopsy and RP specimens was 88.9%. Overall AUC was 0.872 when applied to the validation data set. Nomogram predictions of significant upgrading were within 7.5% of an ideal nomogram. *Conclusions*. Nearly one-fifth of Japanese patients with prostate cancer will be significantly upgraded. Our nomogram seems to provide considerably accurate predictions regardless of minor variations in pathological assessment when applied to Japanese patient populations.

## 1. Introduction

Pretreatment prostate-specific antigen (PSA) level, Gleason score, and pathological stage are generally recognized as significant predictors of biochemical recurrence in patients with clinically localized prostate cancer treated by radical prostatectomy (RP) [1]. A finding of high-grade disease in RP specimens is an adverse prognostic factor, and such tumors are significantly more likely to progress than organ-confined cancers. In addition, this finding is associated with a greater risk of positive surgical margins, further decreasing the likelihood of long-term cancer control. Determining whether a patient has high-grade disease is thus important for treatment selection and prognosis [2].

Chun et al. developed and validated a model predicting Gleason sum upgrading from biopsy to final pathology using clinical variables (PSA level, clinical stage, and biopsy Gleason sum) [3]. That model relies on three readily available clinical variables, all of which are significant uni- and multivariate predictors of biopsy Gleason sum upgrading. Based on the importance of the concept of Gleason sum upgrading in decision making for prostate cancer, we previously performed a formal external validation using a fully independent data set in a contemporary cohort of two Japanese institutions [4]. Unfortunately, our results did not suggest that accurate predictions may be expected when using this nomogram across different racial patient populations. Development of a nomogram predicting the probability of biopsy Gleason sum upgrading in a large multi-institutional cohort among Japanese patients thus appears essential.

## 2. Material and Methods

Clinical and pathological data were prospectively gathered from 837 consecutive patients at two centers (Department of Urology in the Graduate School of Medicine at Chiba University, Chiba (*n* = 327) and Division of Urology at Chiba Cancer Center, Chiba (*n* = 510)). Of these, 71 patients were excluded because of missing data.

Analyses targeted 766 evaluable patients assessed with ≥10 biopsy cores. All men had biopsy-confirmed, clinically localized prostate cancer, and all underwent RP between January 2003 and December 2009. Patients treated with neoadjuvant hormonal therapy were excluded, as the nomogram is not applicable in these men.

Clinical stage was assigned by the attending urologist according to the 2002 TNM system. Under transrectal ultrasound (TRUS) guidance, 10–16 needle cores were obtained. Pretreatment PSA levels were measured before a digital rectal examination (DRE) and TRUS. Biopsy Gleason sum was assigned by pathologists from each center. All RP specimens were processed according to the Stanford protocol and graded according to the Gleason system [5].

Significant upgrading was defined as a biopsy Gleason sum changing from ≤6 to ≥7 or from 7 to ≥8, according to previous reports by King [6] and King and Long [7]. For both patient cohorts, the same predictors, that is, PSA level, primary and secondary biopsy Gleason score, and clinical stage, were used in uni- and multivariate logistic regression models addressing the rate of significant Gleason sum upgrading between biopsy and RP pathology. Coefficients of multivariate logistic regression models were then used to develop a nomogram predicting the probability of significant Gleason sum upgrading, using the data from one Japanese institution: the Division of Urology at Chiba Cancer Center, Chiba (*n* = 508). The variables were selected for the final multivariate model by forward stepwise selection. In addition, we utilized the bootstrapping method to correct for overfit and the bias-corrected coefficients obtained from multivariate analysis to construct the final nomogram. Accuracy of the nomogram was quantified using the receiver operating characteristics (ROC) curve. 

**Figure 1:**
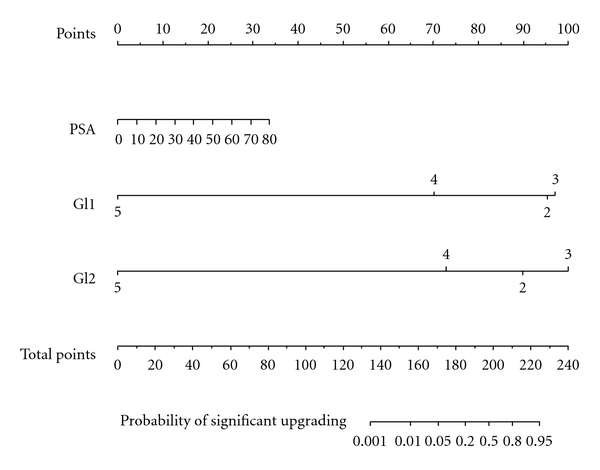
Nomogram based on 508 patients treated at Chiba Cancer Center, for predicting significant Gleason sum upgrading between biopsy and radical prostatectomy. PSA: prostate-specific antigen (ng/mL); Gl1: primary biopsy Gleason score; Gl2: secondary biopsy Gleason score. To obtain the nomogram-predicted probability of significant biopsy upgrading, locate the patient values at each axis, draw a vertical line to the “Points” axis to determine how many points are attributed to each variable value; total the points for all variables, and locate the sum on the “Total Points” line to assess the individual probability of significant biopsy Gleason sum upgrading on the Probability of Significant Upgrading line.

Validation data representing men treated with RP were obtained from another Japanese institution: the Department of Urology in the Graduate School of Medicine at Chiba University, Chiba (*n* = 258). To determine the nomogram-predicted probability of significant Gleason sum upgrading, we applied the nomogram (Figure 1) to all 258 observations. Accuracy of the nomogram was then quantified using the area under the curve (AUC) for external validation. The extent of over- or underestimation relative to the observed rate of significant upgrading was explored graphically using nonparametric Loess smoothing plots. All tests were two sided with a significance level set at *P* < .05.

## 3. Results

Table 1 lists the clinical and pathological characteristics of patients included in this study, and data were stratified for participating institutions. Pretreatment PSA levels were 2.5–79.7 ng/mL. Clinical stages T1c and T2 were recorded in 685 patients (89.4%). Among all men, 578 (75.5%) showed a biopsy Gleason sum of 6 or 7.

In the Chiba Cancer Center dataset (508 men), concordance between biopsy and RP Gleason sum was recorded in 258 (50.8%). Upgrading was recorded in 104 men (20.5%), whereas 146 (28.7%) were downgraded. These data also indicate that 69 patients (13.6%) were upgraded from biopsy Gleason sum ≤6 to pathologic Gleason sum ≥7. The rate of upgrading from biopsy Gleason sum 7 to pathologic Gleason sum ≥8 was 4.5% (*n* = 23). The overall rate of significant upgrading from biopsy to pathologic Gleason sum was 18.1% (92 patients). Conversely, Gleason sum decreased from ≥8 to ≤7 in 82 men (16.1%) and from 7 to ≤6 in 36 (7.1%). Stratified according to institutions, agreement between Gleason biopsy and final pathology was more frequent in the Chiba University data set (146 men, 56.6%) than in that from Chiba Cancer Center (50.8%). Significant upgrading was more frequent for Chiba University (64 men, 24.8%) than for Chiba Cancer Center (92, 18.1%). We also investigated temporal changes in the rate of significant Gleason sum upgrading for two institutions. Although no significant correlation was found, a trend toward a decrease in the rate of significant upgrading since 2006 was seen. 

Table 2 shows uni- and multivariate logistic regression models for PSA, clinical stage, and primary and secondary biopsy Gleason scores with corresponding uni- and multivariate predictive accuracy estimates. Clinical stage was not associated with significant upgrading in univariate analysis (*P* = .131) and was excluded for multivariate analyses. In univariate analyses, primary and secondary biopsy Gleason scores were highly significant predictors of significant Gleason sum upgrading (*P* < .001 and *P* = .002, resp.). Of all predictors, secondary biopsy Gleason score (AUC = 0.784) represented the most informative predictor, followed by primary biopsy Gleason score (AUC = 0.712) and PSA (AUC = 0.569). In multivariate analyses, all variables except for clinical stage were highly significant (*P* ≤ .001). Multivariate 200 bootstrap-corrected predictive accuracy was 88.9% and exceeded the most informative univariate predictor, namely secondary biopsy Gleason score (78.4%). Figure 1 shows the regression coefficient-based nomogram. High PSA values as well as low primary and/or secondary biopsy Gleason scores are risk factors for significant Gleason sum upgrading at final pathology.

**Figure 2:**
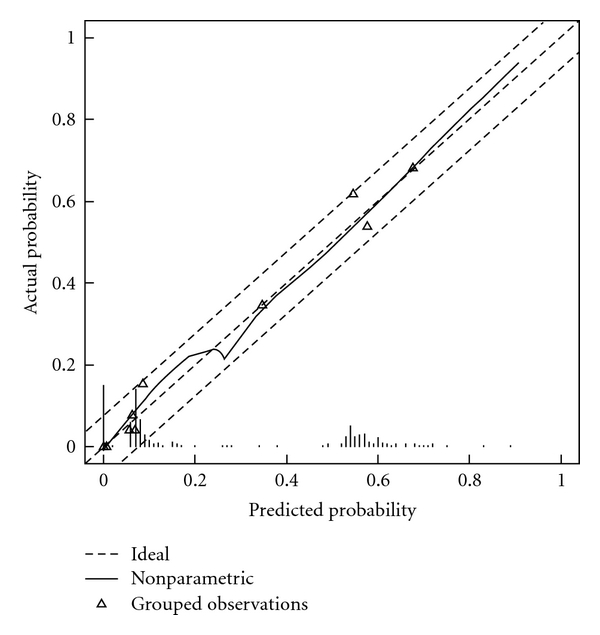
Calibration plot for external validation cohort. The *x*-axis shows the prediction calculated using the nomogram, and the *y*-axis gives observed rates of significant Gleason sum upgrading for patients in the validation cohort. Dashed line indicates reference line, where an ideal nomogram would lie. Solid line indicates performance of the nomogram applied to the validation cohort. The solid line is close to the dashed line of the ideal nomogram and is always within the 7.5% margin of error.

Figure 2 illustrates how predictions of the nomogram are compared with actual probabilities for the validation data (258 men). The *x*-axis represents nomogram predictions, and the *y*-axis represents the observed rate of significant Gleason upgrading for patients in the validation cohort. Accuracy of the nomogram was 87.2% (confidence interval, 82.7–91.7%). The dashed 45° line represents the performance of an ideal nomogram, where predicted outcome would correspond perfectly with actual outcome. The performance of our nomogram is plotted as the solid line. The dotted lines represent a 7.5% margin of error, and the nomogram calibration plot demonstrated virtually ideal predictions. The rate of predicted significant Gleason upgrading closely paralleled the observed rate of Gleason upgrading, nearly corresponding to the 45° line and always within the 7.5% margin of error. The correspondence seen between actual and ideal nomogram predictions suggests good calibration of the nomogram in the validation cohort.

## 4. Discussion

Biopsy upgrading has important clinical implications in terms of watchful waiting, surgery, and radiotherapy (RT) candidates [8–10]. Most reported biopsy Gleason sums are either 6 or 7, and these Gleason sums are at greatest risk of being upgraded. However, tools have previously been unavailable for reliably and accurately predicting this phenomenon. Previous reports have indicated that with more extended biopsy schemes, the risk of upgrading decreases [8, 11] due to higher sampling density and more accurate evaluation of the pathological biopsy. Extended biopsy schemes (≥10 cores) might affect the rate of and ability to predict biopsy Gleason sum upgrading [12]. As a result, ≥14 needle cores are currently obtained in our institutions [13].

King [6] and King and Long [7] defined significant Gleason sum upgrading as a Gleason sum increase either from ≤6 to ≥7 or from 7 to ≥8 between biopsy and RP specimens. They distinguished between any upgrading and significant upgrading and suggested that significant upgrading represents a clinically meaningful entity. Predicting the rate of significant upgrading would be much more clinically meaningful, since these three categories represent pathologically and clinically different diseases. A preparative nomogram predicting the probability of significant Gleason sum upgrading was developed among Western populations [14]. Given the utility of the concept, creation of a new prediction tool based on a modern, Japanese-only cohort and aimed at predicting significant upgrading represents a worthwhile goal.

These findings are important as a first substantial depiction of the rate of significant Gleason sum upgrading in a Japanese contemporary cohort. Several applications of these findings can be considered. For example, the choice of interstitial brachytherapy might be reconsidered in men who are at greater risk of biopsy Gleason sum upgrading. Similarly, neoadjuvant hormonal therapy might be considered if radiotherapy is contemplated. Finally, among surgical candidates, the risk of significant Gleason sum upgrading might contribute to different considerations regarding the extent of neurovascular bundle resection and the implications of positive surgical margins. However, the decision of what level of risk is required for more aggressive therapy remains controversial.

Chun et al. indicated that the rate of upgrading decreased over time [3]. We also investigated temporal changes in the rate of significant Gleason sum upgrading and found no significance. However, a trend toward a decreased rate of significant upgrading over time since 2006 was apparent. This decrease may be due to the impact of the 2005 International Society of Urological Pathology (ISUP) modified Gleason grading system [15]. A shift towards a higher Gleason sum on biopsy might also have occurred after the ISUP consensus [16].

Prostate cancer is one of the most common cancers among Western populations, and incidence is increasing in Asia, although considerable differences in incidence and biological aggressiveness remain between Western and Asian populations [17]. Epidemiological and genetic differences in prostate cancers exist between patients in Japan and the United States, and p53 gene mutational analysis, which often provides information about etiological factors, has revealed clear differences in p53 gene mutational spectra between Japanese and Western cases [18]. Differences in hormone levels in various racial/ethnic groups have been suggested to account for part of the differences in prostate cancer risk. Racial/ethnic differences in the intraprostatic testosterone/dihydrotestosterone conversion ratio would provide important support for the hypothesis that differences in the enzymatic activity of 5a-reductase within the prostate gland can explain most of the racial/ethnic differences in prostate cancer risk [19–21].

We have previously performed a formal external validation of a preparative nomogram predicting the probability of Gleason sum upgrading developed among Western populations, using a fully independent data set in a contemporary cohort of two Japanese institutions [4]. The nomogram provided reasonably accurate predictions regardless of minor variations in pathological assessment but could not necessarily be considered accurate when applied to Japanese patient populations. Our previous results suggested that development of a nomogram predicting the probability of biopsy Gleason sum upgrading in a large multi-institutional cohort among Japanese patients is essential.

We are the first to develop multivariate models to predict significant Gleason sum upgrading between biopsy and RP in Japanese populations. Our current model was 88.9% accurate in predicting the probability of significant Gleason sum upgrading. To date, no other models capable of accurately predicting the rate of significant upgrading are available for Japanese patients. Consequently, this model represents the only alternative to clinical ratings of the probability of significant Gleason sum upgrading. We have therefore tested the performance of the nomogram in an external validation dataset, and overall AUC was 0.87. Individual treatment centers in this study differed with respect to patient selection, extracapsular extension measurement, and follow up assessment. Furthermore, no centralized review of pathology was performed. For the purposes of nomogram validation, such heterogeneity is desirable to gain insights into how the nomogram will perform across varied settings [22]. The nomogram was consistently accurate at both centers, with AUC ranging from 0.87 to 0.89. Our nomogram thus seems to provide reasonably accurate predictions regardless of minor variations in pathological assessment.

Clear limitations exist to this study. We included 10–16 core biopsy data in the cohort, but the difference in rate of upgrading was not significant between these biopsy regimens according to the current data [14]. However, biopsy schemes that rely on taking even more cores might be associated with a lower rate of biopsy Gleason sum upgrading [23–25]. In addition to the small population size, the level of experience of pathologists could also affect the findings. Finally, model accuracy could potentially be improved by integrating additional predictor variables, for example, the level of expertise of the pathologist, or existing biomarkers [26]. If the ISUP modified Gleason grading system or central pathology diagnosis system was introduced, this nomogram should be more useful for daily clinical practice. Despite these limitations, our model represents an important contribution concerning the rate of significant Gleason sum upgrading between biopsy and final pathology.

## 5. Conclusions

Significant Gleason sum upgrading between biopsy and final pathology represents an important consideration in treatment decision making, even in most contemporary patients. Our nomogram was 88.9% accurate in predicting the probability of significant Gleason sum upgrading, and seems to provide accurate predictions regardless of minor variations in pathological assessment when applied to Japanese patient populations.

## Figures and Tables

**Table 1:** Descriptive characteristics of subgroups according to institutions.

Variable		Chiba Cancer Center	Chiba University
*n*		508	258
Age (years)	Mean	66.902	65.054
	SD	4.975	5.253
	Median	67	65
	Min	52	49
	Max	78	76

PSA (ng/mL)	Mean	13.977	11.616
	SD	12.194	9.732
	Median	9.755	8.420
	Min	2.588	2.450
	Max	79.710	72.000

Clinical stage (%)	T1c	169 (33.3)	180 (69.8)
	T2a	172 (33.9)	32 (12.4)
	T2b	89 (17.5)	15 (5.8)
	T2c	12 (2.4)	16 (6.2)
	T3	66 (13.0)	15 (5.8)

Biopsy Gleason primary (%)	≤3	318 (62.6)	172 (66.7)
	4	167 (32.9)	80 (31.0)
	5	23 (4.5)	6(2.3)

Biopsy Gleason secondary (%)	≤3	197 (38.8)	128 (49.6)
	4	227 (44.7)	112 (43.4)
	5	84 (16.5)	18 (7.0)

Biopsy Gleason sum (%)	≤6	123 (24.2)	91 (35.3)
	7	248 (48.8)	116 (45.0)
	8	58 (11.4)	31 (12.0)
	9	74 (14.6)	18 (7.0)
	10	5 (1.0)	2 (0.8)

Pathological Gleason primary (%)	≤3	327 (64.4)	151 (58.5)
	4	155 (30.5)	104 (40.3)
	5	26 (5.1)	3 (1.2)

Pathological Gleason secondary (%)	≤3	209 (41.1)	114 (44.2)
	4	241 (47.4)	119 (46.1)
	5	58 (11.4)	25 (9.7)

Pathological Gleason sum (%)	≤6	93 (18.3)	44 (17.1)
	7	332 (65.4)	176 (68.2)
	8	21 (4.1)	11 (4.3)
	9	60 (11.8)	27 (10.5)
	10	2 (0.4)	0 (0.0)

Significant upgrading Gleason sum (%)		92 (18.1)	64 (24.8)

**Table 2:** Uni- and multivariate logistic regression models predicting significant Gleason sum upgrading.

Predictors	Univariate predictive	Univariate model		Multivariate model	
	accuracy	OR	*P*	OR	*P*
Preoperative PSA	0.569	1.020	.025	1.047	<.001
Clinical stage	NA				
1c		1.000			
2a		0.978	.934	NA	NA
2b		0.715	.334	NA	NA
2c		0.000	.983	NA	NA
3		0.528	.131	NA	NA
Biopsy Gleason primary	0.712				
2		1.000			
3		0.250	<.001	1.210	.677
4		0.041	<.001	0.064	<.001
5		0.000	.983	0.000	.992
Biopsy Gleason secondary	0.784				
2		1.000			
3		1.491	.435	3.050	.041
4		0.189	.002	0.156	.001
5		0.000	.98	0.000	.986
Predictive accuracy				0.889	

OR: odds ratio; PSA: prostate-specific antigen; NA: not assessed.

## Abbreviations and Acronyms

RP: Radical prostatectomy
AUC: Area under the receiver operating characteristic curve
PSA: Prostate-specific antigen
TRUS: Transrectal ultrasound
DRE: Digital rectal examination
ROC: Receiver operating characteristics
RT: Radiotherapy
ISUP: International Society of Urological Pathology.
